# Relationship of Gaming Disorder with parenting based on low affection-communication and personality trait of neuroticism in adolescents

**DOI:** 10.3389/fpsyg.2023.1147601

**Published:** 2023-04-27

**Authors:** Francesc Rodríguez-Ruiz, María Isabel Marí-Sanmillán, Ana Benito, Francisca Castellano-García, Marta Sánchez-Llorens, Isabel Almodóvar-Fernández, Gonzalo Haro

**Affiliations:** ^1^Department of Mental Health, Consorci Hospitalari Provincial de Castelló, Castelló de la Plana, Spain; ^2^TXP Research Group, Universidad Cardenal Herrera-CEU, CEU Universities, Castelló de la Plana, Spain; ^3^Department of Educational Sciences, Universidad Cardenal Herrera-CEU, CEU Universities, Castelló de la Plana, Spain; ^4^Torrente Mental Health Unit, Hospital General de Valencia, Valencia, Spain; ^5^Mislata Mental Health Unit, Hospital de Manises, Manises, Spain; ^6^Nursing Unit Predepartmental, Universitat Jaume I, Castelló de la Plana, Spain

**Keywords:** Gaming Disorder, parenting, personality traits, adolescents, neuroticism, affection-communication

## Abstract

**Background:**

Gaming Disorder is increasingly common in adolescents. We aimed to evaluate the relationship between parenting, personality traits, and Gaming Disorder.

**Methods:**

An observational and cross-sectional study in six secondary schools of Castelló, obtaining a final sample of 397 students.

**Results:**

Adolescents with Gaming Disorder had lower scores in Adolescent Affection-Communication (*F* = 8.201; *p* < 0.001), Father’s Warmth (*F* = 3.459; *p* = 0.028), and Father’s Acceptance/Involvement (*F* = 5.467; *p* = 0.003), and higher scores in Mother’s Revoking Privileges (*F* = 4.277; *p* = 0.034) and Father’s Indifference (*F* = 7.868; *p* = 0.002) than healthy participants. Male sex was a risk factor for Gaming Disorder (OR = 12.221; *p* = 0.004), while Adolescent Affection-Communication (OR = 0.908; *p* = 0.001) and Agreeableness (OR = 0.903; *p* = 0.022) were protective factors. Data modeling described the protective effect that Adolescent Affection-Communication had on Gaming Disorder, which was both directly (*B* = -0.20; *p* < 0.001) and indirectly mediated by Neuroticism (*B* = -0.20; *p* < 0.001), while Neuroticism itself was a risk factor for Gaming Disorder (*B* = 0.50; *p* < 0.001).

**Conclusion:**

These results reflect that Parental style with low affection and communication was directly and indirectly related to the Gaming Disorder, as well as male sex and personality trait of Neuroticism.

## Introduction

Adolescence is a stage of life involving dizzying and radical changes in physical, cognitive, emotional, and social development, in which young adults try to adapt and seek balance within themselves and with society. This period is especially characterized by vulnerability, with those going through it being particularly sensitive to social and environmental models, thereby making it a critical moment for the appearance of both risky and addictive behaviors (Latimer and Zur, 2010). In this context, there is currently some level of social alarm as the result of an increase in excessive substance use and in Gaming Disorder (GD) (Vinet and Faúndez, 2012; López-Caneda et al., 2014). Despite being two different types of addictions, their coexistence is frequent because they both entail similar biological mechanisms (Echeburúa et al., 2009; De Sola et al., 2013). In addition, certain personality variables, understood as persistent patterns of perceiving, thinking, and relating to others, seem to facilitate or predispose individuals to these behaviors (Fantin, 2006).

The problematic use of videogames is an increasingly frequent risky behavior present in the adolescent population. The prevalence of GD in adolescents has increased from 6.1% in 2018 to 7.1% in 2021 (Delegación del Gobierno para el Plan Nacional sobre Drogas, 2021), with these rates being similar to other studies which reported 4.25% in China (Liang et al., 2021) and 8.2% in an international cohort (Porter et al., 2010). According to the Spanish Association of Video Games, 15.9 million people (54.1% men and 45.9% women) were gamers in Spain in 2020 (Asociación Española de Videojuegos, 2020), with this study highlighting the fact that between 68 and 72% of people aged 6–24 years played videogames. Regarding the prevalence in relation to sex, the prevalence increased with age in both sexes, with a range of 53.0–62.4% for men and 29.9–42.3% for women. Adolescents reported playing almost daily (5.8% of boys and 1.3% of girls). Liao et al. (2020) found that being male was significantly related to GD, possibly due to gender differences in online device use. A recent study (Sánchez-Llorens et al., 2021), with high school students, showed that there was a higher proportion of boys with GD. Thus, the probability of GD increased if the subject was male. For this reason, gender was a strong predictor of GD because men are more likely to engage in video game use and to be categorized as more problem gamers than women (Bouna-Pyrrou et al., 2018, Krossbakken et al., 2018).

Consequently, GD, which referred to both offline and online games, was included as a formal diagnosis in the 11th edition of the International Classification of Diseases. Diagnosis includes three negative conditions caused by the misuse of videogames: (1) lack of control of gaming behavior in terms of its initiation, frequency, intensity, duration, completion, and the context in which games are played; (2) the increased priority given to games over other vital interests and daily activities; and (3) maintenance or escalation of the behavior despite being aware of its negative consequences (World Health Organization [WHO], 2018). Although there is controversy over the creation of the Internet Gaming Disorder (IGD) and GD diagnoses, arguing that it could pathologize healthy players and create generalized panic, in addition to the fact that, having used Substance Use Disorder (SUD) diagnostic criteria, an overdiagnosis of GD could be incurred (Billieux et al., 2019). Furthermore, previous literature has generally not distinguished between online vs offline gaming when assessing IGD.

Personality traits can be conceived as habitual patterns of attitude, behavior, emotion, and thought which are relatively stable over time, differ across individuals and influence behavior. The Big Five model of personality conceives of personality as a result of the interactions among five broad personality dimensions: neuroticism, openness to experience, conscientiousness, extraversion, and agreeableness (Gervasi et al., 2017). Certain personality traits have been related to GD, especially low conscientiousness (Sánchez-Llorens et al., 2021), impulsivity, lower self-control/self-regulation, sensation, stimulation, and/or novelty seeking, a tendency toward boredom, risky behavior, hostility/animosity, and enhanced levels of aggression (Paulus et al., 2018). GD has also been related with neuroticism, low self-esteem, alexithymia, and dysfunctions in emotion regulation (Bonnaire and Baptista, 2019). Other personality traits that may predispose individuals to GD are introversion or shyness, decreased openness, agreeableness, resourcefulness, irritability and anxiety, and narcissistic, avoidant, and schizoid traits (Paulus et al., 2018). In contrast, perseverance/grit (Bouna-Pyrrou et al., 2018) and self-directedness may be protective factors against GD (Brand et al., 2016). In the study carried out by Liao et al. (2020), the results indicated that the GD was significantly associated with personality traits such as neuroticism and conscientiousness, a result consistent with previous published literature on the subject (Yan et al., 2014; Yao et al., 2014; Bonnaire and Baptista, 2019; Sánchez-Llorens et al., 2021). The significant relationship between neuroticism and GD is because highly neurotic people see the real world as a threat and often turn to digital worlds, where they feel safe. Consequently, negative emotionality correlates positively with gaming problems and is strongly related to neuroticism (Müller et al., 2014) as long as it is possible to conceptualize problematic online gaming as a maladaptive coping strategy that may serve to reduce tension as a mood modifier (Gervasi et al., 2017). Moreover, adolescents with greater self-control and self-management of time, which also are related to the conscientiousness personality trait, have a lower correlation with GD (Chen et al., 2020), as long as people with low conscientiousness who are less persistent in pursuing personal aims and pay less attention to duties of everyday life may find computer games particularly attractive and do not think through the consequences of engaging in activities excessively. Additionally, decreased agreeableness indicates a higher trend toward competition which may reinforce the game behavior, introvert people which lack social skills may find in computer games a way to search for social contacts in controlled virtual environments and low openness may tend to stick to their gaming behavior instead of exploring new activities (Gervasi et al., 2017; Şalvarlı and Griffiths, 2019)

The family is the main context of socio-emotional development during childhood and adolescence and so parenting practices or parental socialization styles contribute to the acquisition of skills that prevent addictive behaviors (Koning et al., 2018). Regarding parental socialization styles, two main dimensions are considered: affection-communication (related to the emotional tone and behavior of parents toward their children, by which children feel that they are loved and feel accepted as individuals within the family) and the control-structure of parents/guardians (related to the degree of intensity or type of influence that parents exert on their children’s behavior) (Maccoby and Martin, 1983; Benito et al., 2019). Analyzing the most studied parenting practices for their specific relationship with the problematic use of videogames, we find three categories of parental behavior: active mediation (which means having conversations about the use of the Internet and sharing experiences), restrictive mediation (the authorization to use particular online applications) and social co-use (viewing the screen together) (Nathanson, 1999; Valkenburg et al., 1999; Nikken and Jansz, 2014). In this direction, the empirical literature, consisting mainly of cross-sectional studies, seems to be quite inconsistent regarding the role of restrictive parental mediation. While some defend the effectiveness of restrictive parental measures to regulate the use of video games (Martins et al., 2017; Koning et al., 2018), others find that this type of practice encourages their problematic use (Shin and Ismail, 2014; Benrazavi et al., 2015). It is the same for the role of active mediation. In the published literature on the subject, data show that the family environment can be both a risk factor and a protective factor in relation to adolescent gaming behaviors. Thus, parental care expressed as empathy, closeness, emotional warmth, and affection was associated with lower scores in game results (Floros et al., 2013). In this line, Bonnaire and Phan (2017) concluded that the parents’ attitude about the use of games, as well as family functioning, are factors that exert a strong influence on the appearance of GD. Prevention strategies should include psychoeducation in order to understand the concept of GD, teach time management skills, stress management and self-control techniques, develop social relationships, set gaming time limits and identify alternative activities (King et al., 2018) which were demonstrated to increase perceptions of risks associated with excessive use of video games, the factors related to GD and of the characteristics of an GD gamer (Bonnaire et al., 2019b). Those prevention polices would be stronger if they included education for parents on how the games and Internet works, including practical tips for monitoring and setting limits (King et al., 2018). As pathological gamers tend to come from less warm and cohesive families with low parental support and adaptability, they may benefit from family-based interventions since by involving parents in therapy in order to close the emotional distance between parents and adolescents that may improve the communication and by changing the negative perception of the adolescent who comes to treatment and avoiding the notion of sole responsibility to allow making better therapeutic alliances with both parents and adolescents (Bonnaire et al., 2019a).

While some studies document familial protective factors of online gaming in adolescents (Estévez et al., 2017; Kim et al., 2018; Liang et al., 2021; Macur and Pontes, 2021), others showed that other important characteristics such as personality, mental health, and other psychological factors may affect online gaming in teenagers (Spilkova et al., 2017). For some adolescents with low levels of emotion regulation or poor emotion regulation skills, playing video games is a maladaptive strategy used to cope with individual and familial difficulties so excessive video gaming can be considered as an escape strategy (Blasi et al., 2019; Bonnaire et al., 2019a). It has been found that emotional warmth of both parents has an influence on adolescents’ GD being mediated by time management trait, so parents interacting with adolescents in a warm and accepting way may favor self-control, self-efficacy, and autonomy in order to improve time management and prevent developing of GD (Chen et al., 2020). However, very little literature has integrated parenting and personality traits with the development of GD in adolescents while also considering possible addictions to substances. Perhaps because it is a more complex relationship, we have not found studies that also add the role of gender to the equation. However, it has been suggested that sons and daughters are socialized differently, and that the impact of parenting on behavior problems is different for boys and girls (Barnett and Scaramella, 2013). Since sex differences have also been found in every phase of addiction (acute reinforcing effects, transition from occasional to compulsive use, withdrawal-associated negative affective states, craving, and relapse) (Becker and Chartoff, 2019) we believe it is interesting to study the role of sex in the relationship of these variables.

Thus, the objectives of this current study were to evaluate the parental socialization styles related to GD in adolescents; differences in parenting practices received by adolescents with no addiction (NA), those exhibiting Excessive Gaming (EG) tendencies, or with GD; the relationship of GD and adolescent personality traits, psychopathology, and behavioral problems; and the role of sex in this relationship. Our hypotheses were that: parenting based on affection-communication is negatively related to GD; some personality traits are positively, and others negatively related to GD; psychopathology and behavior are related to GD; and sex moderates these relationships.

## Materials and methods

### Participants

This was an observational and cross-sectional study. The sample comprised 397 students (and their primary caregivers) in the third or fourth years of Compulsory Secondary Education (CSE). They were all from five private subsidized schools and one public school in the province of Castellón (Spain) that were selected by purposive sampling. With the G*Power 3.1.9.4 program, it was calculated that the sample needed to perform ANOVA with four groups, effect size 0.25, alpha 95% and power 80% was 180 subjects.

### Measures

The Questionnaire of Experiences Associated with Videogames (CERV) in its original Spanish initialism (Chamarro et al., 2014), was used to assesses the problematic use of videogames. The CERV comprises 17 items and its cut-off point is ≥26. The Negative Consequences subscale and the Dependence and Avoidance subscale presented a Cronbach alpha of 0.869 and 0.861, respectively, with an overall Cronbach alpha score of 0.912.

The Game Addiction Scale for Adolescents (GASA) (Lloret et al., 2017) was used to assess GD, and consists of 7 items with a cut-off point ≥4. The Spanish adaptation presented a Cronbach alpha reliability of 0.81, which was consistent with the findings published by the original authors (Lemmens et al., 2009).

The Alcohol Use Disorders Identification Test (AUDIT) (Babor et al., 2001), allowed us to identify excessive alcohol consumption in our cohort. The AUDIT contains 10 questions with a cut-off point of ≥6 in women and ≥8 in men. Its internal consistency indices were usually around 0.80 (Allen et al., 1997). Finally, the instrument showed a sensitivity of 57–59% and a specificity of 91–96% (Álvarez et al., 2001).

The Car, Relax, Alone, Forget, Family/Friends, Trouble (CRAFFT) test (Rial et al., 2019), which comprises six dichotomous items (yes/no) and has a cut-off of ≥2 positive items, was used to screen for the risky use of alcohol and other substances in adolescents. This tool presented an internal consistency of 0.74, with a sensitivity of 74.4% and a specificity of 96.4%.

The Problem Oriented Screening Instrument for Teenagers (POSIT) (Araujo et al., 2018) was employed to assess the risky consumption of alcohol and other drugs in adolescents. POSIT presents 17 dichotomous items and has a cut-off point of ≥2 positive items. The Spanish version presents an internal consistency of 0.82, sensitivity 94.3%, and specificity 83.9%.

The TXP Parenting Questionnaire (Benito et al., 2019) is subdivided into two questionnaires: the TXP-A which is applied to adolescents and the TXP-C applied to the primary caregiver. The TXP-A consists of 29 items and two factors (affection-communication and control-structure), while the TXP-C comprises 16 items and two factors: affection-communication and prosocial values. The overall Cronbach alpha (reliability) of the TXP was 0.87 and the test-retest value was 0.94.

The Parental Socialization Scale, or ESPA-29 in its Spanish acronym (Musitu and García, 2004), assesses parental socialization styles through 212 items that evaluate the adolescent’s perception of the way their parents/guardians act in 29 different situations. It is based on two axes of socialization: Acceptance/Involvement (i.e., expression of reactions of approval and affection when children behave in accordance with family norms) and Strictness/Imposition (a socialization style used when children behave in a way that differs from the norms of family functioning). The internal consistency of the ESPA-29 was high and varied between 0.82 and 0.94 depending on the factors (Iglesias and Romero Triñanes, 2009).

The Big Five Personality Test for Children and Adolescents (BFQ-NA) (Barbaranelli et al., 2013) is an adaptation of the Big Five Personality Model. The internal consistency of the overall scale was 0.86 and by subscales it was as follows: Consciousness = 0.87, Agreeableness = 0.82, Neuroticism = 0.83, Extraversion = 0.76, and Openness = 0.75 (Soto et al., 2011).

The Behavior Assessment System for Children (BASC) (González et al., 2004; Reynolds and Kamphaus, 2004) contains 5 components that can be used together or individually. In this current study we used the Self-Report (S3) completed by adolescents and a questionnaire for Parents (P3). The internal consistency of the global dimensions of the BASC were between 0.76 and 0.96, with a mean value of 0.91. S3 provides data from clinical scales and 4 global dimensions: School Maladjustment (SMC), Clinical Maladjustment (CMC), Personal Adjustment (PAC), and the Emotional Symptoms Index (ESI). The P3 questionnaire measures maladaptive behaviors, which allowed us to obtain values for Externalizing problems, Internalizing problems, and Adaptive skills, as well as a Behavioural Symptoms Index (BSI).

### Procedure

After authorization by the participating educational centers, a letter was sent to the guardians of the students in the third and fourth years of CSE to request authorization for their children to participate in this study. Once the authorization was obtained, the questionnaires were filled out by the students for an hour and a half during school hours on two consecutive days. The surveys were completed between October and December 2018 with the supervision of two psychologists. The parents/guardians of participating students received the questionnaires by post and returned them completed to the school. Neither the adolescents nor their relatives received compensation of any type for their collaboration.

Four groups were formed: participants with a score above the CERV and the GASA cut-off point (GD; *n* = 27), with a score above the CERV cut-off point and below the GASA cut-off point (EG; *n* = 47) which would be made up of those people with an excessive use of video games without reaching a significant functional impairment that would allow it to be defined as a Gaming Disorder, as has been described in previous research (Kuss and Griffiths, 2012), in order to distinguish between excessive gamers and pathological gamers, those with a (SUD) (a score above the cut-off point for 2 of the AUDIT, CRAFFT, and POSIT questionnaires; *n* = 37), and healthy participants (*n* = 171). Individuals who scored above the cut-off point on only one substance questionnaire were excluded because we considered this insufficient evidence of the presence of a substance addiction, although this result was not considered healthy. Of these excluded subjects, 44.6% had neither GD nor EG, 42.9% had EG, and 12.4% had GD. We also decided to exclude participants with a dual pathology (GD and SUD), as well as any participants with EG and SUD. These two groups were eliminated as they were not independent of the SUD, GD, and EG groups. Also, for presenting sample sizes much smaller than the rest of the groups, since, although recent evidence shows that F is robust to the difference in group size, there is still evidence that in ANOVA an excessively large difference in the sample sizes between groups led to reduced power (Liang et al., 2020).

### Statistical analysis

SPSS software (v23, IBM Corp., Armonk, NY, USA) was employed to check compliance with the assumptions of the statistical tests used and analyse the relationships between the study variables by using chi-squared (categorical variables) and ANOVA (quantitative variables) tests, considering the results significant when *p* < 0.05. Once the comparisons between the four groups were made, to explore the variables specifically related to the healthy-excessive gaming-pathological gaming progression, these comparisons were repeated comparing these three groups (NA, EG, and GD). Substance use disorders were evaluated only to control for this variable, which could distort the results referring to the objectives of the study. Since the SUD is more studied, the rest of the analyzes were carried out excluding this group to specifically explore the variables related to gaming. We used multinomial logistic regression by a forward stepwise method to study whether the independent variables of parental socialization, personality, psychopathology, and behavior, and the sociodemographic variables that were significant in ANOVA and chi-squared tests allowed the dependent variables of GD and EG to be predicted (using as reference category NA). To avoid multicollinearity problems, we run linear regression procedures and successive logistic regression models until finding the model that contained the uncorrelated independent variables with the highest predictive power. Finally, a model was constructed using PROCESS v3.4 (Hayes, 2017) for SPSS to evaluate the hypothesis that parenting is a protective factor against GD. The PROCESS model that best fit the data was number 14: *X* = AAC, *Y* = GD, *M* = Neuroticism, *W* = Sex.

### Ethics

The principles of the Declaration of Helsinki and the Convention of the Council of Europe (World Medical Association [WMA], 2013) were always met. The confidentiality of the participants and their data was guaranteed according to the General Data Protection Regulation (GDPR) law of May 2016 (European Parliament and the Council of the European Union, 2016). The students and guardians included in this study signed their informed consent prior to participation. The overall study protocol was authorized by the Ministry of Education, Research, Culture, and Sport (CN00A/2018/25/S), the ethics committee at the Cardenal Herrera-CEU University (CEI18/112), and by the Research commission of the Consorci Hospitalari Provincial de Castelló (3-16/12/19).

## Results

Of the 397 participants, 43.1% (*n* = 171) had NA, 11.8% (*n* = 47) showed EG, 6.8% (*n* = 27) had GD, 9.3% (*n* = 37) exhibited a SUD, 3.8% (*n* = 15) had an EG and SUD, and 1.5% (*n* = 6) had a GD and SUD. Comorbid participants (who showed both EG and a SUD or both GD and a SUD) were excluded from the following analyses. Tables 1–3 and Supplementary Table 1 show the descriptions and significant differences between the four groups included in terms of the sociodemographic data and TXP, ESPA-29, BFQ-C, and BASC questionnaire results.

**TABLE 1:** Descriptive statistics of the sociodemographics of the overall sample and by the no addiction, Excessive Gaming, Gaming Disorder, and Substance Use Disorder groups.

	Total *n* = 397 *n* (%)/M (SD)	NA *n* = 171 *n* (%)/M (SD)	EG *n* = 47 *n* (%)/M (SD)	GD *n* = 27 *n* (%)/M (SD)	SUD *n* = 37 *n* (%)/M (SD)	χ^2^ (P); ES (CTR)/F (P); ES *Post-hoc*: (*P*)	Effect size
Sex						**59.99 (< 0.001); 0.46**	0.46
Female	226 (57.1)	**122 (74.4)**	6 (3.7)	10 (6.1)	26 (15.9)	(**5.6/-6.9/-2.4**/1.6)	
Male	170 (42.9)	48 (41)	**41 (35)**	**17 (14.5)**	11 (9.4)	(**-5.6/6.9/2.4**/-1.6)	
Age in years	14.82 (0.74)	14.73 (0.70)	14.62 (0.64)	14.59 (0.69)	15.00 (0.67)	**2.68 (0.047); 0.02**	0.02
School year						**7.85 (0.049); 0.16**	0.16
Third year of CSE	169 (42.7)	83 (62.9)	23 (17.4)	16 (12.1)	10 (7.6)	(0.8/0.3/1.3/**-2.6**)	
Fourth year of CSE	227 (57.3)	87 (58.4)	24 (16.1)	11 (7.4)	**27 (18.1)**	(-0.8/-0.3/-1.3/**2.6**)	
Repeated courses (school years)						6.41 (0.379)	0.11
None	281 (80.5)	123 (59.1)	36 (17.3)	23 (11.1)	26 (12.5)		
1 repeated course	46 (13.2)	16 (61.5)	1 (3.8)	2 (7.7)	7 (26.9)		
2 repeated courses	22 (6.3)	7 (58.3)	2 (16.7)	1 (8.3)	2 (16.7)		
Number of siblings of the student	2.10 (0.96)	2.03 (0.99)	2.08 (0.86)	2.42 (1.27)	2.17 (0.79)	1.28 (0.283)	0.01
People with whom they live						10.61 (0.101)	0.14
Both parents	270 (77.1)	109 (58.6)	34 (18.3)	18 (9.7)	25 (13.4)		
Father or mother only	73 (20.9)	35 (62.5)	5 (8.9)	6 (10.7)	10 (17.9)		
Others	7 (2)	1 (25)	1(25)	2 (50)	0 (0)		

CTR, corrected typified residuals; those under -1.96 or over 1.96 were considered significant. The groups from among the categorical variables in which the CTRs were significant are shown in bold; EG, Excessive Gaming; ES, effect size; GD, Gaming Disorder; M, average; n, sample; NA, no addiction; SD, standard deviation; SUD, Substance Use Disorder; χ^2^, Pearson chi-squared test; The variables that were significant in chi-squared and ANOVA tests (p < 0.05) are shown in bold. Pearson chi-squared test’s effect size = Cramer’s V: 0-1. ANOVA’s effect size = partial eta squared: 0.01 small, 0.06 medium, 0.14 large. Differences between the variables were tested using chi-squared and ANOVA tests.

**TABLE 2:** Descriptive statistics of parenting of the overall sample and by the no addiction, Excessive Gaming, Gaming Disorder, and Substance Use Disorder groups.

	Total *n* = 397 M (SD)	NA *n* = 171 M (SD)	EG *n* = 47 M (SD)	GD *n* = 27 M (SD)	SUD *n* = 37 M (SD)	F (P); ES *Post-hoc*: (P)	Effect size
**TXP Parenting Questionnaire**							
Adolescent: affection–communication	82.79 (14.49)	86.78 (12.83)	85.09 (14.50)	75.37 (16.23)	72.97 (18.75)	**12.41 (< 0.001); 0.12** **0.008 (NA > GD)** **0.001 (NA > SUD)** **0.011 (EG > SUD)**	0.29
Adolescent: control–structure	35.08 (5.88)	35.86 (5.64)	35.56 (6.18)	34.85 (6.18)	31.92 (6.63)	**4.49 (0.004); 0.04** **0.002 (NA > SUD)** **0.032 (EG > SUD)**	0.05
Caregivers: prosocial values	19.40 (1.42)	19.62 (0.94)	18.81 (2.83)	19.27 (1.03)	19.07 (1.59)	**3.60 (0.014); 0.04**	0.10
Caregivers: affection–communication	54.82 (7.51)	56.39 (7.16)	52.91 (9.12)	52.86 (9.48)	52.95 (8.97)	**2.97 (0.033); 0.04**	0.11
**ESPA-29**							
Mother’s reasoning	2.97 (0.70)	3.03 (0.68)	3.00 (0.71)	2.91 (0.65)	2.82 (0.81)	0.80 (0.494)	0.04
Mother’s warmth	2.99 (0.78)	3.04 (0.79)	3.04 (0.80)	2.93 (0.72)	2.73 (0.84)	1.31 (0.271)	0.03
Mother’s detachment	1.34 (0.37)	1.26 (0.28)	1.33 (0.35)	1.39 (0.41)	1.50 (0.47)	**4.38 (0.005); 0.06**	0.13
Mother’s indifference	1.76 (0.73)	1.65 (0.68)	1.90 (0.88)	1.86 (0.70)	1.94 (0.80)	2.11 (0.100)	0.04
Mother’s physical punishment	1.06 (0.15)	1.04 (0.13)	1.07 (0.15)	1.13 (0.23)	1.10 (0.16)	**3.55 (0.015); 0.04**	0.02
Mother’s revoking privileges	1.72 (0.64)	1.63 (0.62)	1.86 (0.68)	2.00 (0.73)	1.70 (0.65)	**2.85 (0.038); 0.03**	0.01
Mother’s verbal scolding	2.57 (0.67)	2.54 (0.72)	2.59 (0.69)	2.80 (0.58)	2.72 (0.52)	1.23 (0.301)	0.007
Mother’s acceptance/ involvement	3.24 (0.49)	3.30 (0.48)	3.22 (0.41)	3.15 (0.47)	3.04 (0.59)	2.02 (0.114)	0.07
Mother’s strictness/imposition	1.79 (0.41)	1.75 (0.42)	1.83 (0.45)	1.97 (0.46)	1.84 (0.36)	1.82 (0.144)	0.006
Father’s reasoning	2.74 (0.77)	2.81 (0.79)	2.95 (0.74)	2.41 (0.70)	2.64 (0.69)	2.46 (0.064)	0.11
Father’s warmth	2.76 (0.86)	2.89 (0.82)	2.88 (0.86)	2.36 (0.99)	2.59 (0.85)	**2.98 (0.033); 0.04**	0.11
Father’s detachment	1.46 (0.50)	1.38 (0.52)	1.39 (0.49)	1.51 (0.47)	1.62 (0.38)	1.84 (0.142)	0.09
Father’s indifference	1.96 (0.82)	1.73 (0.73)	2.08 (0.79)	2.35 (0.87)	2.27 (0.79)	**7.39 (< 0.001); 0.09** **0.004 (NA < GD)** **0.004 (NA < SUD)**	0.12
Father’s physical punishment	1.05 (0.16)	1.04 (0.15)	1.01 (0.04)	1.10 (0.19)	1.13 (0.27)	**3.87 (0.010); 0.05**	0.15
Father’s revoking privileges	1.62 (0.59)	1.56 (0.61)	1.79 (0.65)	1.82 (0.54)	1.64 (0.58)	2.08 (0.104)	0.05
Father’s verbal scolding	2.38 (0.64)	2.33 (0.67)	2.54 (0.73)	2.52 (0.62)	2.49 (0.55)	1.27 (0.286)	0.02
Father’s acceptance/involvement	3.00 (0.59)	3.13 (0.57)	3.06 (0.51)	2.65 (0.61)	2.82 (0.55)	**4.85 (0.003); 0.08** **0.006 (NA > GD)**	0.17
Father’s strictness/imposition	1.68 (0.37)	1.64 (0.39)	1.78 (0.41)	1.81 (0.38)	1.75 (0.36)	2.11 (0.101)	0.04

EG, Excessive Gaming; ES, effect size; GD, Gaming Disorder; M, average; n, sample; NA, no addiction; SD, standard deviation; SUD, Substance Use Disorder; variables with significant ANOVA test results (*p* < 0.05) are shown in bold. Effect size = partial eta squared: 0.01 small, 0.06 medium, 0.14 large. Differences between variables were tested by ANOVA.

**TABLE 3:** Descriptive statistics of personality traits of the overall sample and by the no addiction, Excessive Gaming, Gaming Disorder, and Substance Use Disorder groups.

	Total *n* = 397 M (SD)	NA *n* = 171 M (SD)	EG *n* = 47 M (SD)	GD *n* = 27 M (SD)	SUD *n* = 37 M (SD)	F (P); ES *Post-hoc*: (P)	Effect size
Conscientiousness	54.00 (9.63)	57.04 (8.99)	54.60 (9.98)	48.33 (9.42)	48.00 (8.90)	**14.55 (< 0.001); 0.13** **< 0.001 (NA > GD)** **< 0.001 (NA > SUD)** **0.026 (EG > GD)** **0.007 (EG > SUD)**	0.13
Openness	55.92 (9.46)	57.96 (9.39)	57.06 (10.28)	52.37 (10.05)	50.32 (8.19)	**8.34 (< 0.001); 0.08** **0.024 (NA > GD)** **< 0.001 (NA > SUD)** **0.007 (EG > SUD)**	0.08
Extraversion	50.72 (10.00)	51.19 (9.69)	49.96 (9.39)	45.26 (11.78)	48.00 (11.17)	**3.28 (0.021); 0.03** **< 0.024 (NA > GD)**	0.03
Agreeableness	52.97 (9.48)	54.49 (9.30)	54.34 (9.70)	45.37 (8.09)	49.05 (8.83)	**10.27 (< 0.001); 0.10** **< 0.001 (NA > GD)** **0.007 (NA > SUD)** **< 0.001 (EG > GD)** **0.046 (EG > SUD)**	0.10
Neuroticism	50.07 (11.35)	47.45 (11.26)	46.87 (9.04)	53.96 (12.31)	60.32 (10.80)	**16.36 (< 0.001); 0.15** **0.023 (NA < GD)** **< 0.001 (NA < SUD)** **0.039 (EG < GD)** **< 0.001 (EG < SUD)**	0.15

EG, Excessive Gaming; ES, effect size; GD, Gaming Disorder; M, average; n, sample; NA, no addiction; SD, standard deviation; SUD, Substance Use Disorder; Variables with significant ANOVA test results (*p* < 0.05) are shown in bold. Effect size = partial eta squared: 0.01 small, 0.06 medium, 0.14 large. Differences between variables were tested by ANOVA.

We studied the differences between parenting practices, personality, psychopathology, and the behavior of adolescents with EG, GD, or NA (excluding those with SUD). Tables 4, 5 show the differences between the NA, EG, and GD groups. Regarding parental socialization, participants with GD had lower scores in Adolescent Affection-Communication (AAC), Father’s Warmth, and Father’s Acceptance/Involvement and higher scores in Mother’s Revoking Privileges and Father’s Indifference than individuals with NA, while those showing EG had higher AAC, Father’s Reasoning, and Father’s Acceptance/Involvement scores than in the GD group as well as higher Father’s Indifference than the NA group. Regarding personality traits, the participants with GD presented higher Neuroticism and lower scores in Conscientiousness, Openness, Extraversion, and Agreeableness than individuals with NA. In turn, participants with GD obtained higher scores in Neuroticism and lower scores in Conscientiousness and Agreeableness than those with GA. No differences were found in personality traits between the EG and NA groups.

**TABLE 4:** Differences between the no addiction, Excessive Gaming, and Gaming Disorder groups according to ANOVA analysis (F[p]); ES.

	NA		
	**Parenting**	**Personality traits**	**Behavior and psychopathology**
**EG**	Father’s indifference: 7.868 (0.029); 0.08^EG^		Conduct problems: 2.678 (0.036); 0.02 ^NA^
**GD**	Adolescent affection–communication: 8.201 (< 0.001); 0.06 ^NA^ Father’s warmth: 3.459 (0.028); 0.03 ^NA^ Father’s acceptance/involvement: 5.467 (0.003); 0.07 ^NA^ Mother’s revoking privileges: 4.277 (0.034); 0.04^GD^ Father’s indifference: 7.868 (0.002); 0.08 ^GD^	Conscientiousness: 10.706 (< 0.001); 0.08 ^NA^ Openness: 3.935 (0.015); 0.03 ^NA^ Extraversion: 4.230 (0.011); 0.03 ^NA^ Agreeableness: 11.588 (< 0.001); 0.08 ^NA^ Neuroticism: 4.428 (0.013); 0.03 ^GD^	Interpersonal relationships: 11.184 (0.005); 0.08 ^NA^ Relationship with parents: 9.938 (0.006); 0.07 ^NA^ Self-esteem: 6.682 (0.032); 0.05 ^NA^ Self-reliance: 11.304 (0.005); 0.08 ^NA^ Personal adjustment: 16.992 (0.001); 0.12 ^NA^ Negative attitude toward school: 9.072 (< 0.001); 0.07 ^GD^ Negative attitude toward teachers: 9.523 (0.003); 0.07 ^GD^ Atypicality: 3.021 (0.039); 0.02 ^GD^ Locus of control: 12.092 (< 0.001); 0.09 ^GD^ Social stress: 21.823 (< 0.001); 0.15 ^GD^ Anxiety: 3.317 (0.029); 0.02 ^GD^ Depression: 18.814 (< 0.001); 0.13 ^GD^ Sense of inadequacy: 10.816 (0.007); 0.08 ^GD^ Clinical maladjustment: 8.557 (< 0.001); 0.06 ^GD^ School maladjustment: 9.612 (< 0.001); 0.07 ^GD^ ESI: 19.648 (0.001); 0.14 ^GD^ Attention problems: 6.130 (0.002); 0.05 ^GD^

EG, Excessive Gaming; ES, effect size; ESI, Emotional Symptom Index; GD, Gaming Disorder; NA, no addiction; the name of the group (GD, EG or NA) that scored highest in Tukey *post-hoc* tests for homogeneous variance or in Games–Howell *post-hoc* significance comparison tests for non-homogeneous variance (*p* < 0.05) is shown after each variable in superscript.

**TABLE 5:** Differences between Excessive Gaming and Gaming Disorder according to ANOVA analysis (*F*[*p*]); ES.

	EG		
	**Parenting**	**Personality traits**	**Behavior and psychopathology**
**GD**	Adolescent affection–communication: 8.201 (0.010); 0.06^EG^ Father’s reasoning: 3.139 (0.038); 0.03 ^EG^ Father’s acceptance/Involvement: 5.467 (0.043); 0.07 ^EG^	Conscientiousness: 10.706 (0.015); 0.08 ^EG^ Agreeableness: 11.588 (< 0.001); 0.08 ^EG^ Neuroticism: 4.428 (0.022); 0.03 ^GD^	Interpersonal relationships: 11.184 (0.013); 0.08 ^EG^ Relationship with parents: 9.938 (0.004); 0.07 ^EG^ Self-reliance: 11.304 (0.021); 0.08 ^EG^ Personal adjustment: 16.992 (0.001); 0.12 ^EG^ Negative attitude toward school: 9.072 (0.005); 0.07 ^GD^ Locus of control: 12.092 (0.001); 0.09 ^GD^ Social stress: 21.823 (< 0.001); 0.15 ^GD^ Depression: 18.814 (0.002); 0.13 ^GD^ Sense of inadequacy: 10.816 (0.038); 0.08 ^GD^ Clinical maladjustment: 8.557 (0.006); 0.06 ^GD^ School maladjustment: 9.612 (0.021); 0.07 ^GD^ ESI: 19.648 (0.003); 0.14 ^GD^

EG, Excessive Gaming; ES, effect size; ESI, Emotional Symptom Index; GD, Gaming Disorder; the name of the group (GD or EG) that scored highest in Tukey *post-hoc* tests for homogeneous variance or in Games–Howell *post-hoc* significance comparison tests for non-homogeneous variance (*p* < 0.05) is shown after each variable in superscript.

Supplementary Table 2 shows the unadjusted logistic regression model, while Table 6 shows the model adjusted for age, parenting, personality, behavior, and psychopathology. The presence of GD was predicted by Male Sex with an Adjusted Odds Ratio (OR) of 12.221, as well as AAC (OR = 0.908), and Agreeableness (OR = 0.903). Furthermore, GA was also predicted by Male Sex (OR = 27.645). The separate ORs of each questionnaire are shown in the Supplementary material (Supplementary Tables 3–11).

**TABLE 6:** Odds ratio of the multiple logistic regression model (using no addiction as the reference category) adjusted by age, parenting, personality, behavior, and psychopathology by a forward stepwise method to predict the dependent variables of Excessive Gaming and Gaming Disorder.

Dependent variables	Independent variables	OR (95% CI)	*p*-value
EG	Sex*	**27.645** **(7.121–107.318)**	**<0.001**
	Adolescent affection–communication	0.975 (0.915–1.039)	0.431
	Agreeableness	1.004 (0.943–1.070)	0.896
GD	Sex*	**12.221** **(2.275–65.635)**	**0.004**
	**Adolescent affection–communication**	**0.908** **(0.857–0.962)**	**0.001**
	**Agreeableness**	**0.903** **(0.827–0.986)**	**0.022**

CI, confidence interval; EG, Excessive Gaming; GD, Gaming Disorder; OR, odds ratio; The variables with a significant OR in the multiple logistic regression model (*p* < 0.05) are shown in bold.

*Belonging to the male sex.

**FIGURE 1:**
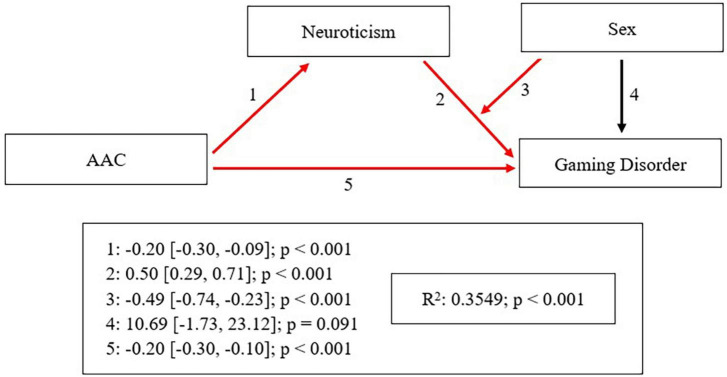
Explanatory model between parenting, personality, and Gaming Disorder. Significant relationships are shown in red. AAC, Adolescent affection-communication.

Finally, Figure 1 shows the model that describes the protective effect of AAC on GD, both directly (the more AAC, the less GD) and indirectly, with the latter being mediated by Neuroticism, for which it was also a protective factor (the more AAC, the less Neuroticism). Indeed, Neuroticism was a risk factor for GD (the more Neuroticism, the more GD) and was moderated by Sex: it is mainly in boys where this relationship between Neuroticism and GD occurs, that is, Neuroticism is a risk factor for GD mainly in boys.

## Discussion

We fulfilled the main objective of this study: to identify the relationship between GD and parenting, personality traits, psychopathology, and behavioral problems in adolescents, while also exploring the differences between individuals with NA and those showing EG or a GD. The main contribution of this work is that the perception by adolescents that their relationship with their parents or guardians presented Affection-Communication behaved as a protective factor against the development of GD. Another important scientific contribution, which can also be applied in prevention and treatment programs, is the different role of Affection-Communication between the EG and GD groups, which could mean that a parental relationship based on affection and communication can prevent an adolescent with excessive videogames use from developing an addiction.

Indeed, previous studies have shown the relevance of communication between parents/guardians and children in relation to the development of GD (Estévez et al., 2017). Liang et al. (2021) showed that a high Parent-Adolescent Communication score can help adolescents feel satisfied in their basic psychological needs without having to resort to psychological compensation through the addictive use of videogames. Another study also revealed higher odds of GD among adolescents with greater difficulties in communicating with their parents/guardians (Macur and Pontes, 2021). The study in Korean adolescents by Kim et al. (2018) revealed that Affection and Communication with the father reduced the risk of developing GD, without the same influence being noted for this factor in the mother. Similarly, our study revealed a relationship between GD and low Warmth, Acceptance/Involvement, and high Indifference from the father, while the mother impacted GD through Revoking Privileges. Therefore, psychotherapy that improves affection and communication can lead to a decrease in GD symptoms, as was demonstrated by a study of an intervention program for adolescents with GD that included a module of family communication (Torres-Rodríguez et al., 2018a) or by a randomized controlled trial of Family therapy to reduce GD (Nielsen et al., 2021).

However, Xu et al. (2015) found that maternal attachment factors were more significantly associated with addiction and the onset of GD than paternal attachment. This suggests that when adolescents feel in a safe parental environment, they tend to present fewer risky behaviors, with their family being perceived as a pivotal factor in determining their ability to develop skills for coping with life’s difficulties, with such learning serving to reinforce emotional regulation (Estévez et al., 2017). Therefore, if an individual feels unlovable and neglected and has developed a negative self-concept because of negative relationships during childhood, videogames may offer safer environments for adolescents to develop their self-esteem and identity because they can create alternative virtual identities and use them as a shelter or escape (Estévez et al., 2017).

Regarding personality, various publications have shown a relationship between GD and personality traits (Sánchez-Llorens et al., 2021). Focusing on the Big-Five Personality Traits, our study showed that low Agreeableness as well as Neuroticism acted as a risk-factors for the development of GD as a result of low Affection-Communication, with the same differences also being found with respect to adolescents with GD. Furthermore, our data also revealed that adolescents with GD presented lower levels of Conscientiousness, Openness, and Extraversion than those with NA. The relationship between low Agreeableness and GD can be explained because these individuals tend to compete rather than cooperate, as usually occurs in the types of online videogames that require high levels of competitiveness.

Teenagers with high Neuroticism tended to perceive the real world as more threatening and so they often took refuge in the virtual world of videogames in the search for a safer and more controllable environment. Adolescents with low Conscientiousness levels presented lower scores for self-directedness and attention to everyday obligations, which was directly related to SMC, represented by a negative attitude toward school and teachers, itself a risk for developing GD. In addition, these adolescents tended to be disorganized and unstructured and so finding an environment with a structure and clear rules such as in a videogame may have been attractive to them.

Low extraversion and GD could be related because of a lack of social skills, low sociability (Festl et al., 2013), and problems with interpersonal relationships. This means that these adolescents may have compensated for difficulties in making and maintaining friendships in the real world by interacting with other people online through videogames where they could form new relationships and even have a sense of belonging and group identity (Gallimberti et al., 2016; Estévez et al., 2017). Another possible interpretation is because the use of videogames is usually a solitary activity (Gallimberti et al., 2016). Finally, low Openness could be related to the development of GD because participants with this trait tend to cling to play behavior rather than exploring novel activities (Müller et al., 2014; Torres-Rodríguez et al., 2018b; González-Bueso et al., 2020). Perhaps for this same reason, unlike previous studies (Hu et al., 2017), we found no relationship between Sensation Seeking and GD.

Regarding psychopathology and behavioral problems, there is evidence of a relationship between GD and symptoms of anxiety, depression, suicidal ideation, Attention-Deficit Hyperactivity Disorder (ADHD), autism spectrum disorder, and obsessive-compulsive disorder (Andreassen et al., 2016; Torres-Rodríguez et al., 2018b). The findings in our study were similar, with GD being related to CMC and emotional symptoms such as anxiety and depression that could explain the use of videogames as a maladaptive form of emotional self-regulation. We also found that GD was related to attention problems, which may be because ADHD is a risk factor for addiction on its own, but also because a person with ADHD may begin to seek rewards and show hyperfocus through behaviors such as becoming absorbed in videogames, perhaps even using them as a form of “self-medication.”

Playing videogames requires a series of cognitive functions such as attention, visual processing, visuospatial memory, and executive control (Mathews et al., 2019). Our data show that a sense of inadequacy, low self-esteem, and low self-reliance are often found in adolescents with GD, which may be related to their need to improve their self-esteem through game features such as feedback, promotions, scoring, accomplishments, anonymity, creation of personal social identities, comfortable expression of self, or interaction with other players (Toker and Baturay, 2016). Regarding such behavior, some studies such as the one by Kim et al. (2018) have related aggressive behavior to presenting a higher risk of developing GD. However, in our study we did not find differences regarding aggressiveness between those with GD and healthy adolescents.

Moreover, like most other studies (Paulus et al., 2018; Macur and Pontes, 2021), we found that male sex was an important predictor of GD. The disparity between genders was attributed in some studies, such as the one by Phan et al., to the differences in videogame preferences between genders, with men preferring strategy, role-playing, action, and fighting genres and women preferring social, puzzle, card, music, dance, educational, and simulation genres (Phan et al., 2012). In other more recent studies such as the one by Dong et al. the difference in risk between genders was related to differences at the neurobiological level, demonstrating greater activation in men in the right striatum, right orbitofrontal cortex, left inferior frontal gyrus, and right middle occipital gyrus, with bilateral decline when exposed to gaming-related cues thereby also generating more craving (Dong et al., 2018).

According to the data obtained in our study, the probability of presenting a video game use disorder seems to be greater in males, as shown in the published literature on the subject (Liao et al., 2020; Sánchez-Llorens et al., 2021). So, gender appears to be a strong predictor of GD. Boys are more likely to engage in video game use and to be categorized as more problem gamers than girls (Sánchez-Llorens et al., 2021). On the other hand, GD is significantly associated with some personality traits, such as neuroticism (Liao et al., 2020). The relationship between neuroticism and GD occurs mainly in boys, precisely because the prevalence of the disorder is higher in this population. In the model proposed in our work, gender moderates the direct relationship between neuroticism and DG, but they do not have a direct effect on DG; thus, the boys who present greater neuroticism will present greater GD. As there are more boys with GD, this aspect may have influenced the result. In this sense, it could be thought that the differences found between GD and SUD may be due to sex, so that, *a posteriori*, we repeat all the analyzes adjusting for sex and age. The results did not vary substantially, maintaining these significant differences.

Regarding the limitations of this study, first, its cross-sectional design implies that causality cannot be inferred based on these data. Second, there is still a lack of consensus regarding the criteria and psychometric instruments required to diagnose GD. In our study, EG and GD were classified using screening questionnaires, which must be considered when making comparisons with other studies and in the extrapolation of results. In addition, the study relies on self-reported metrics, which can originate bias effects and under- or overreporting of behaviors and may therefore result in social desirability bias. Another limitation is that the videogame genres used by the participants was not considered even though this factor may be relevant when identifying specific risk factors for subpopulations of adolescents with GD. However, the exclusion of patients with SUD from the final analyses, but above all having considered problematic or risky substance use as an exclusion criterion (score on a single screening questionnaire), should be considered as a selection bias take into account when generalizing the results. Moreover, we have decided to exclude SUD from the analyzes since it is a more studied topic and thus specifically explore the variables related to GD. Furthermore, our study cohort consisted of a convenience sample since the secondary schools included were not randomly chosen. To finalize the limitations, it should be noted that the educational centers did not provide reliable information regarding the total number of students in the target population, which has prevented us from establishing a response rate of students who participated. In addition, the requirements related to the protection of personal data do not allow us to know the reasons for non-participation.

Regarding the strengths of this study, we evaluated variables both from the perspectives of adolescents and of their guardians, which helped to provide us with a broader vision of the outcomes. We also included metrics of parental socialization, personality, and psychopathological variables in the same study. In addition, we differentiated two profiles of videogame users, one with an excessive but not addicted gaming profile and the other with an addiction profile with greater problems at the paternal-filial, personality, and psychopathological levels. It is also noteworthy that most of the effect sizes of the significant differences between the groups are medium-large, which is especially striking since gaming is a complex multi-causal phenomenon.

## Conclusion

In this work, the parental style of low affection and communication was directly related to the GD. In addition, low affection and communication was also indirectly related to high neuroticism in adolescents, which in turn, was linked to GD. Furthermore, male sex was also related to GD. However, only male sex was shown to be related to EG in adolescents and so neither parental style nor personality traits seemed to be relevant to this diagnosis.

## Data availability statement

The raw data supporting the conclusions of this article will be made available by the authors, without undue reservation.

## Ethics statement

The studies involving human participants were reviewed and approved by the Ministry of Education, Research, Culture, and Sport Ethics Committee at the Cardenal Herrera-CEU University Research Commission of the Consorci Hospitalari Provincial de Castelló. Written informed consent to participate in this study was provided by the participants’ legal guardian/next of kin.

## Author contributions

MM-S, AB, and GH conceptualized and designed the study. GH obtained the founding sources and ethical authorizations. MM-S, IA-F, and FC-G collected the data. FR-R and MS-L performed quality assurance for all the data and coordinated database activities. FR-R and AB performed the data analysis and interpretation. FR-R drafted the manuscript. AB and GH supervised the study and elaboration of all the manuscript. All authors assisted with subsequent drafts, were responsible for reviewing and approving the final version of the manuscript, had full access to all data in the study, took responsibility for the integrity of the data and the accuracy of the data analysis, and contributed and approved the final manuscript.

## References

[B1] Allen J. P. Litten R. Z. Fertig J. B. Babor T. (1997). A review of research on the alcohol use disorders identification test (AUDIT). *Alcohol. Clin. Exp. Res.* 21 613–619. 9194913

[B2] Álvarez S. Gallego P. Latorre C. Bermejo F. (2001). Papel del Test AUDIT (Alcohol Use Disorders Identification Test) para la detección de consumo excesivo de alcohol en Atención Primaria. *Medifam* 11 553–557.

[B3] Andreassen C. S. Billieux J. Griffiths M. D. Kuss D. J. Demetrovics Z. Mazzoni E. (2016). The relationship between addictive use of social media and video games and symptoms of psychiatric disorders: a large-scale cross-sectional study. *Psychol. Addict. Behav.* 30 252–262. 10.1037/adb0000160 26999354

[B4] Araujo M. Golpe S. Braña T. Varela J. Rial A. (2018). Validación psicométrica del POSIT para el cribado del consumo de riesgo de alcohol y otras drogas entre adolescentes. *Adicciones* 30 130–139. 10.20882/adicciones.958 28492958

[B5] Asociación Española de Videojuegos. (2020). *La Industria Del Videojuego En España Anuario 2020.* Available online at: http://www.aevi.org.es/web/wp-content/uploads/2021/04/AEVI_Anuario_2020.pdf (accessed May 10, 2021).

[B6] Babor T. F. Higgins-Biddle J. C. Saunders J. B. Monteiro M. G. (2001). *AUDIT: The Alcohol Use Disorders Identification Test. Guidelines for use in Primary Care.* Geneva: World Health Organization.

[B7] Barbaranelli C. Caprara G. Rabasca A. Barrio M. V. Carrasco Ortíz M. Holgado Tello F. P. (2013). *BFQ-NA: Cuestionario “Big Five” de Personalidad Para Niños y Adolescentes: Manual (3a ed. rev.).* Madrid: TEA.

[B8] Barnett M. A. Scaramella L. V. (2013). Mothers’ parenting and child sex differences in behavior problems among African American preschoolers. *J. Family Psychol.* 27 773–783. 10.1037/a0033792 23937420 PMC3981992

[B9] Becker J. B. Chartoff E. (2019). Sex differences in neural mechanisms mediating reward and addiction. *Neuropsychopharmacology* 44 166–183. 10.1038/s41386-018-0125-6 29946108 PMC6235836

[B10] Benito A. Calvo G. Real-López M. Gallego M. J. Francés S. Turbi Á (2019). Creation of the TXP parenting questionnaire and study of its psychometric properties. Creación y estudio de las propiedades psicométricas del cuestionario de socialización parental TXP. *Adicciones* 31 117–135. 10.20882/adicciones.983 29353299

[B11] Benrazavi R. Teimouri M. Griffiths M. D. (2015). Utility of parental mediation model on youth’s problematic online gaming. *Int. J. Ment. Health Addict.* 13 712–727. 10.1007/s11469-015-9561-2

[B12] Billieux J. Flayelle M. Rumpf H. J. Stein D. J. (2019). High involvement versus pathological involvement in video games: a crucial distinction for ensuring the validity and utility of gaming disorder. *Curr. Addict. Rep.* 6 323–330. 10.1007/s40429-019-00259-x

[B13] Blasi M. D. Giardina A. Giordano C. Coco G. L. Tosto C. Billieux J. (2019). Problematic video game use as an emotional coping strategy: evidence from a sample of MMORPG gamers. *J. Behav. Addict.* 8 25–34. 10.1556/2006.8.2019.02 30739460 PMC7044601

[B14] Bonnaire C. Baptista D. (2019). Internet Gaming Disorder in male and female young adults: the role of alexithymia, depression, anxiety and gaming type. *Psychiatry Res.* 272 521–530. 10.1016/j.psychres.2018.12.158 30616119

[B15] Bonnaire C. Liddle H. A. Har A. Nielsen P. Phan O. (2019a). Why and how to include parents in the treatment of adolescents presenting Internet gaming disorder? *J. Behav. Addict.* 8 201–212. 10.1556/2006.8.2019.27 31146552 PMC7044550

[B16] Bonnaire C. Serehen Z. Phan O. (2019b). Effects of a prevention intervention concerning screens, and video games in middle-school students: influences on beliefs and use. *J. Behav. Addict.* 8 537–553. 10.1556/2006.8.2019.54 31537087 PMC7044621

[B17] Bonnaire C. Phan O. (2017). Relationships between parental attitudes, family functioning and Internet gaming disorder in adolescents attending school. *Psychiatry Res.* 255 104–110. 10.1016/j.psychres.2017.05.030 28535475

[B18] Bouna-Pyrrou P. Aufleger B. Braun S. Gattnar M. Kallmayer S. Wagner H. (2018). Cross-sectional and longitudinal evaluation of the social network use disorder and internet gaming disorder criteria. *Front. Psychiatry* 9:692. 10.3389/fpsyt.2018.00692 30627106 PMC6310011

[B19] Brand M. Young K. S. Laier C. Wölfling K. Potenza M. N. (2016). Integrating psychological and neurobiological considerations regarding the development and maintenance of specific Internet-use disorders: an Interaction of Person-Affect-Cognition-Execution (I-PACE) model. *Neurosci. Biobehav. Rev.* 71 252–266. 10.1016/j.neubiorev.2016.08.033 27590829

[B20] Chamarro A. Carbonell X. Manresa J. M. Munoz-Miralles R. Ortega-González R. Lopez-Morron M. R. (2014). El Cuestionario de Experiencias Relacionadas con los Videojuegos (CERV): un instrumento para detectar el uso problemático de videojuegos en adolescentes españoles [The Questionnaire of Experiences Associated with Video games (CERV): an instrument to detect the problematic use of video games in Spanish adolescents]. *Adicciones* 26 303–311. 25578001

[B21] Chen I. H. Lee Z. H. Dong X. Y. Gamble J. H. Feng H. W. (2020). The influence of parenting style and time management tendency on internet gaming disorder among adolescents. *Int. J. Environ. Res. Public Health* 17:9120. 10.3390/ijerph17239120 33291336 PMC7730530

[B22] De Sola J. Rubio G. Rodríguez de Fonseca F. (2013). La impulsividad: >Antesala de las adicciones comportamentales? *Health Addict.* 13 145–155. 10.21134/haaj.v13i2.211

[B23] Delegación del Gobierno para el Plan Nacional sobre Drogas (2021). *ESTUDES 2021. Encuesta Sobre el Uso de Drogas en Enseñanzas Secundarias en España.* Available online at: https://pnsd.sanidad.gob.es/ca/profesionales/sistemasInformacion/sistemaInformacion/pdf/ESTUDES_2021_Presentacion_enWeb.pdf (accessed November 18, 21021).

[B24] Dong G. Wang L. Du X. Potenza M. N. (2018). Gender-related differences in neural responses to gaming cues before and after gaming: implications for gender-specific vulnerabilities to Internet gaming disorder. *Soc. Cogn. Affect. Neurosci.* 13 1203–1214. 10.1093/scan/nsy084 30272247 PMC6234325

[B25] Echeburúa E. Labrador F. Becoña E. (2009). *Adicción a las Nuevas Tecnologías en Adolescentes y Jóvenes.* Madrid: Pirámide.

[B26] Estévez A. Jáuregui P. Sánchez-Marcos I. López-González H. Griffiths M. D. (2017). Attachment and emotion regulation in substance addictions and behavioral addictions. *J. Behav. Addict.* 6 534–544. 10.1556/2006.6.2017.086 29280395 PMC6034944

[B27] European Parliament and the Council of the European Union (2016). *Regulation (EU) 2016/679 of the European parliament and of the council of 27 April 2016 on the protection of natural persons with regard to the processing of personal data and on the free movement of such data, and repealing Directive 95/46/EC (general data protection regulation)*. Strasbourg: European Parliament.

[B28] Fantin M. B. (2006). Perfil de personalidad y consumo de drogas en adolescentes escolarizados. *Adicciones* 18 285–292. 10.20882/adicciones.346

[B29] Festl R. Scharkow M. Quandt T. (2013). Problematic computer game use among adolescents, younger and older adults. *Addiction* 108 592–599. 23078146 10.1111/add.12016

[B30] Floros G. D. Siomos K. Fisoun V. Geroukalis D. (2013). Adolescent online gambling: the impact of parental practices and correlates with online activities. *J. Gambl. Stud.* 29 131–150. 10.1007/s10899-011-9291-8 22271406

[B31] Gallimberti L. Buja A. Chindamo S. Rabensteiner A. Terraneo A. Marini E. (2016). Problematic use of video games and substance abuse in early adolescence: a cross-sectional study. *Am. J. Health Behav.* 40 594–603. 10.5993/AJHB.40.5.6 27561862

[B32] Gervasi A. M. La Marca L. Costanzo A. Pace U. Guglielmucci F. Schimmenti A. (2017). Personality and internet gaming disorder: a systematic review of recent literature. *Curr. Addict. Rep.* 4 293–307. 10.1007/s40429-017-0159-6

[B33] González J. Fernández S. Pérez E. Santamaría P. (2004). *Adaptación Española del Sistema de Evaluación de la Conducta en Niños y Adolescentes: BASC.* Madrid: TEA Ediciones.

[B34] González-Bueso V. Santamaría J. Oliveras I. Fernández D. Montero E. Baño M. (2020). Internet gaming disorder clustering based on personality traits in adolescents, and its relation with comorbid psychological symptoms. *Int. J. Environ. Res. Public Health* 17:1516. 10.3390/ijerph17051516 32111070 PMC7084409

[B35] Hayes A. F. (2017). *Introduction to Mediation, Moderation, and Conditional Process Analysis: a Regression-Based Approach*, 2 Edn. New York, NY: Guilford Publications, 120–141.

[B36] Hu J. Zhen S. Yu C. Zhang Q. Zhang W. (2017). Sensation seeking and online gaming addiction in adolescents: a moderated mediation model of positive affective associations and impulsivity. *Front. Psychol.* 8:699. 10.3389/fpsyg.2017.00699 28529494 PMC5418345

[B37] Iglesias B. Romero Triñanes E. (2009). Estilos parentales percibidos, psicopatología y personalidad en la adolescencia. *Rev. Psicopatol. Psicol. Clín.* 14 63–77. 10.5944/rppc.vol.14.num.2.2009.4067

[B38] Kim E. Yim H. W. Jeong H. Jo S. J. Lee H. K. Son H. J. (2018). The association between aggression and risk of Internet gaming disorder in Korean adolescents: the mediation effect of father-adolescent communication style. *Epidemiol. Health* 40:e2018039. 10.4178/epih.e2018039 30089352 PMC6232655

[B39] King D. L. Delfabbro P. H. Doh Y. Y. Wu A. M. S. Kuss D. J. Pallesen S. (2018). Policy and prevention approaches for disordered and hazardous gaming and internet use: an international perspective. *Prevent. Sci.* 19 233–249. 10.1007/s11121-017-0813-1 28677089

[B40] Koning I. M. Peeters M. Finkenauer C. van den Eijnden R. (2018). Bidirectional effects of Internet-specific parenting practices and compulsive social media and Internet game use. *J. Behav. Addict.* 7 624–632. 10.1556/2006.7.2018.68 30273047 PMC6426398

[B41] Krossbakken E. Pallesen S. Mentzoni R. A. King D. L. Molde H. Finserås T. R. (2018). A cross-lagged study of developmental trajectories of video game engagement, addiction, and mental health. *Front. Psychol.* 9:2239. 10.3389/fpsyg.2018.02239 30519203 PMC6258776

[B42] Kuss D. J. Griffiths M. D. (2012). Internet and gaming addiction: a systematic literature review of neuroimaging studies. *Brain Sci.* 2 347–374. 10.3390/brainsci2030347 24961198 PMC4061797

[B43] Latimer W. Zur J. (2010). Epidemiologic trends of adolescent use of alcohol, tobacco, and other drugs. *Child Adolesc. Psychiatr. Clin. North Am.* 19 451–464. 10.1016/j.chc.2010.03.002 20682214 PMC6413882

[B44] Lemmens J. S. Valkenburg P. M. Peter J. (2009). Development and validation of a game addiction scale for adolescents. *Media Psychol.* 12 77–95.

[B45] Liang Q. Yu C. Xing Q. Liu Q. Chen P. (2021). The influence of parental knowledge and basic psychological needs satisfaction on peer victimization and internet gaming disorder among Chinese adolescents: a mediated moderation model. *Int. J. Environ. Res. Public Health* 18:2397. 10.3390/ijerph18052397 33804528 PMC7967736

[B46] Liang Q. Yu X. An S. (2020). Nan fang yi ke da xue xue bao. *J. South. Med. Univ.* 40 713–717. 10.12122/j.issn.1673-4254.2020.05.16 32897205 PMC7277317

[B47] Liao Z. Huang Q. Huang S. Tan L. Shao T. Fang T. (2020). Prevalence of internet gaming disorder and its association with personality traits and gaming characteristics among Chinese adolescent gamers. *Front. Psychiatry* 11:598585. 10.3389/fpsyt.2020.598585 33312143 PMC7704426

[B48] Lloret D. Gomis R. Marzo J. Tirado S. (2017). Validación española de la escala de adicción a videojuegos para adolescentes (GASA). *Atención Prim.* 50 350–358. 10.1016/j.aprim.2017.03.015 28939247 PMC6836933

[B49] López-Caneda E. Mota N. Crego A. Velasquez T. Corral M. Holguín S. R. (2014). Anomalías neurocognitivas asociadas al consumo intensivo de alcohol (binge drinking) en jóvenes y adolescentes: una revisión. *Adicciones* 26 334–359. 10.20882/adicciones.39 25578003

[B50] Maccoby E. E. Martin J. A. (1983). “Socialization in the context of the family: parent-child interaction,” in *Handbook of Child Psychology: Formerly Carmichael’s Manual of Child Psychology*, ed. Mussen P. H. (New York, NY: Wiley).

[B51] Macur M. Pontes H. M. (2021). Internet Gaming Disorder in adolescence: investigating profiles and associated risk factors. *BMC Public Health* 21:1547. 10.1186/s12889-021-11394-4 34384394 PMC8361866

[B52] Martins N. Matthews N. L. Ratan R. A. (2017). Playing by the rules: parental mediation of video game play. *J. Family Issues* 38 1215–1238. 10.1177/0192513X15613822

[B53] Mathews C. L. Morrell H. Molle J. E. (2019). Video game addiction, ADHD symptomatology, and video game reinforcement. *Am. J. Drug Alcohol Abuse* 45 67–76. 29874473 10.1080/00952990.2018.1472269

[B54] Müller K. W. Beutel M. E. Egloff B. Wölfling K. (2014). Investigating risk factors for Internet gaming disorder: a comparison of patients with addictive gaming, pathological gamblers and healthy controls regarding the big five personality traits. *Eur. Addict. Res.* 20 129–136. 10.1159/000355832 24247280

[B55] Musitu G. García F. (2004). *ESPA29, Escalas De Estilos De Socialización En La Adolescencia*, 2 Edn. Madrid: TEA ediciones.

[B56] Nathanson A. I. (1999). Identifying and explaining the relationship between parental mediation and children’s aggression. *Commun. Res.* 26 124–143. 10.1177/009365099026002002

[B57] Nielsen P. Christensen M. Henderson C. Liddle H. A. Croquette-Krokar M. Favez N. (2021). Multidimensional family therapy reduces problematic gaming in adolescents: a randomised controlled trial. *J. Behav. Addict.* 10 234–243. 10.1556/2006.2021.00022 33905350 PMC8996793

[B58] Nikken P. Jansz J. (2014). Developing scales to measure parental mediation of young children’s internet use. *Learn. Media Technol.* 39 250–266. 10.1080/17439884.2013.782038

[B59] Paulus F. W. Ohmann S. von Gontard A. Popow C. (2018). Internet gaming disorder in children and adolescents: a systematic review. *Dev. Med. Child Neurol.* 60 645–659. 10.1111/dmcn.13754 29633243

[B60] Phan M. H. Jardina J. R. Hoyle S. Chaparro B. S. (2012). Examining the role of gender in video game usage, preference, and behavior. *Proc. Hum. Fact. Ergon. Soc. Annu. Meet.* 56 1496–1500. 10.1177/1071181312561297

[B61] Porter G. Starcevic V. Berle D. Fenech P. (2010). Recognizing problem video game use. *Aust. N. Z. J. Psychiatry* 44 120–128. 10.3109/00048670903279812 20113300

[B62] Reynolds C. R. Kamphaus R. W. (2004). *BASC: Sistema De Evaluación de la Conducta De Niños y Adolescentes: Manual.* Madrid: TEA.

[B63] Rial A. Kim-Harris S. Knight J. R. Arauji M. Gómez P. Braña T. (2019). Empirical validation of the CRAFFT abuse screening test in a Spanish sample. Validación empírica del CRAFFT abuse screening test en una muestra de adolescentes españoles. *Adicciones* 31 160–169. 10.20882/adicciones.1105 29353300

[B64] Şalvarlı Şİ Griffiths M. D. (2019). Internet gaming disorder and its associated personality traits: a systematic review using PRISMA guidelines. *Int. J. Ment. Health Addict.* 19 1420–1442. 10.1007/s11469-019-00081-6

[B65] Sánchez-Llorens M. Marí-Sanmillán M. I. Benito A. Rodríguez-Ruiz F. Castellano-García F. Almodóvar I. (2021). Personality traits and psychopathology in adolescents with videogame addiction. Rasgos de personalidad y psicopatología en adolescentes con adicción a videojuegos. *Adicciones* [Online ahead of print]. 10.20882/adicciones.1629 34882237

[B66] Shin W. Ismail N. (2014). Exploring the role of parents and peers in young adolescents’ risk taking on social networking sites. *Cyberpsychol. Behav. Soc. Netw.* 17 578–583. 10.1089/cyber.2014.0095 25126969

[B67] Soto G. Ferrándiz C. Sáinz M. Ferrando M. Prieto M. D. Bermejo R. (2011). Características psicométricas del cuestionario de personalidad BFQNA (Big five questionnaire- niños y adolescentes). *Aula Abierta* 39 13–24.

[B68] Spilkova J. Chomynová P. Csémy L. (2017). Predictors of excessive use of social media and excessive online gaming in Czech teenagers. *J. Behav. Addict.* 6 611–619. 10.1556/2006.6.2017.064 29039223 PMC6034940

[B69] Toker S. Baturay M. H. (2016). Antecedents and consequences of game addiction. *Comput. Hum. Behav.* 55 668–679. 10.1016/j.chb.2015.10.002

[B70] Torres-Rodríguez A. Griffiths M. D. Carbonell X. (2018a). The treatment of internet gaming disorder: a brief overview of the PIPATIC program. *Int. J. Ment. Health Addict.* 16 1000–1015. 10.1007/s11469-017-9825-0 30147635 PMC6096606

[B71] Torres-Rodríguez A. Griffiths M. D. Carbonell X. Oberst U. (2018b). Internet gaming disorder in adolescence: psychological characteristics of a clinical sample. *J. Behav. Addict.* 7 707–718. 10.1556/2006.7.2018.75 30264606 PMC6426364

[B72] Valkenburg P. M. Krcmar M. Peeters A. L. Marseille N. M. (1999). Developing a scale to assess three styles of television mediation: “Instructive mediation,” “restrictive mediation,” and “social coviewing.”. *J. Broadcast. Electron. Media* 43 52–66. 10.1080/08838159909364474

[B73] Vinet E. V. Faúndez X. (2012). Consumo de alcohol y drogas en adolescentes evaluado a través del MMPI-A. *Salud Ment.* 35 205–213.

[B74] World Health Organization [WHO] (2018). *11th Edition of the International Classification of Diseases.* Available online at: https://www.who.int/news/item/18-06-2018-who-releases-new-international-classification-of-diseases-(icd-11) (accessed June 18, 2018).

[B75] World Medical Association [WMA] (2013). World medical association declaration of Helsinki: ethical principles for medical research involving human subjects. *JAMA* 310 2191–2194. 10.1001/jama.2013.281053 24141714

[B76] Xu H. Wen L. M. Rissel C. (2015). Associations of parental influences with physical activity and screen time among young children: a systematic review. *J. Obesity* 2015:546925. 10.1155/2015/546925 25874123 PMC4383435

[B77] Yan W. Li Y. Sui N. (2014). The relationship between recent stressful life events, personality traits, perceived family functioning and internet addiction among college students. *Stress Health* 30 3–11. 10.1002/smi.2490 23616371

[B78] Yao M. Z. He J. Ko D. M. Pang K. (2014). The influence of personality, parental behaviors, and self-esteem on Internet addiction: a study of Chinese college students. *Cyberpsychol. Behav. Soc. Netw.* 17 104–110. 10.1089/cyber.2012.0710 24003966 PMC3924803

