# Supplementary material for: Relationship of Gaming Disorder with parenting based on low affection-communication and personality trait of neuroticism in adolescents
Source: Front Psychol. 2023 Apr 27;14:1147601. doi: 10.3389/fpsyg.2023.1147601 (PMC10174293; doi:10.3389/fpsyg.2023.1147601)
Supplement: Supplementary file 1 [file Data_Sheet_1.docx]

Supplementary Material

Relationship of Gaming Disorder with parenting based on low affection-communication and personality trait of neuroticism in adolescents

**Francesc Rodríguez-Ruiz*, María Isabel Marí-Sanmillán, Ana Benito, Francisca Castellano-García, Marta Sánchez-Llorens, Isabel Almodóvar-Fernández, Gonzalo Haro**

*** Correspondence:** Francesc Rodríguez-Ruiz franfranf14@gmail.com

**SUPPLEMENTARY TABLE 1 |** Descriptive statistics of behavior and psychopathology of the overall sample and by the no addiction, Excessive Gaming, Gaming Disorder, and Substance Use Disorder groups. Differences between variables were tested by ANOVA.

|  | Total  n = 397  M (SD) | NA  n = 171  M (SD) | EG  n = 47  M (SD) | GD  n = 27  M (SD) | SUD  n = 37  M (SD) | F (P); ES  Post-hoc: (P) | EFFECT SIZE |
| --- | --- | --- | --- | --- | --- | --- | --- |
| Negative attitude toward school | 50.96 (10.97) | 47.90 (9.70) | 48.96 (9.38) | 56.52 (10.83) | 56.30 (11.45) | **11.37 (< 0.001); 0.11**  **< 0.001 (NA < GD)**  **< 0.001 (NA < SUD)**  **0.011 (EG < GD)**  **0.006 (EG < SUD)** | 0.11 |
| Negative attitude toward teachers | 47.55 (10.08) | 44.92 (8.77) | 47.39 (10.16) | 53.19 (11.17) | 54.05 (10.97) | **13.07 (< 0.001); 0.12**  **0.005 (NA < GD)**  **< 0.001 (NA < SUD)**  **0.029 (EG < SUD)** | 0.10 |
| Sensation Seeking | 49.06 (10.50) | 46.73 (9.48) | 47.26 (11.06) | 47.67 (10.54) | 55.57 (9.95) | **8.20 (< 0.001); 0.08**  **< 0.001 (NA < SUD)**  **0.001 (EG < SUD)**  **0.010 (GD < SUD)** | 0.05 |
| Atypicality | 48.50 (9.74) | 45.70 (8.23) | 46.39 (7.55) | 49.85 (8.71) | 56.03 (9.51) | **16.48 (< 0.001); 0.15**  **< 0.001 (NA < SUD)**  **< 0.001 (EG < SUD)**  **0.020 (GD < SUD)** | 0.14 |
| Locus of Control | 48.18 (11.28) | 44.61 (8.69) | 45.91 (9.63) | 54.00 (11.48) | 55.38 (13.72) | **16.72 (< 0.001); 0.15**  **0.002 (NA < GD)**  **< 0.001 (NA < SUD)**  **0.017 (EG < GD)**  **0.004 (EG < SUD)** | 0.15 |
| Somatisation | 50.81 (11.06) | 49.28 (9.84) | 47.83 (7.93) | 52.30 (12.15) | 58.76 (13.82) | **9.85 (< 0.001); 0.09**  **0.001 (NA < SUD)**  **< 0.001 (EG < SUD)** | 0.09 |
| Social Stress | 50.27 (11.02) | 47.66 (8.82) | 47.61 (9.91) | 60.67 (13.60) | 54.27 (12.19) | **16.36 (< 0.001); 0.15**  **< 0.001 (NA < GD)**  **0.016 (NA < SUD)**  **< 0.001 (EG < GD)**  **0.044 (EG < SUD)** | 0.13 |
| Anxiety | 49.89 (10.47) | 47.88 (10.36) | 48.96 (9.25) | 53.33 (11.09) | 54.03 (10.44) | **5.09 (0.002); 0.05**  **0.002 (NA < SUD)** | 0.03 |
| Depression | 50.74 (12.05) | 47.39 (8.90) | 48.04 (9.66) | 59.74 (14.34) | 57.81 (15.61) | **17.86 (< 0.001); 0.16**  **0.001 (NA < GD)**  **0.002 (NA < SUD)**  **0.003 (EG < GD)**  **0.008 (EG < SUD)** | 0.16 |
| Sense of Inadequacy | 51.34 (11.19) | 48.59 (9.12) | 50.04 (9.80) | 58.26 (14.91) | 58.32 (13.79) | **13.16 (< 0.001); 0.12**  **0.014 (NA < GD)**  **0.001 (NA < SUD)**  **0.016 (EG < SUD)** | 0.14 |
| Interpersonal Relationships | 49.36 (10.27) | 50.79 (9.61) | 50.13 (8.39) | 41.00 (14.40) | 48.70 (9.40) | **7.66 (< 0.001); 0.07**  **0.010 (NA > GD)**  **0.023 (EG > GD)** | 0.08 |
| Relationship with Parents | 49.38 (12.11) | 52.13 (9.90) | 52.74 (6.70) | 43.37 (13.14) | 39.84 (16.79) | **16.90 (< 0.001); 0.15**  **0.012 (NA > GD)**  **0.001 (NA > SUD)**  **0.008 (EG > GD)**  **< 0.001 (EG > SUD)** | 0.19 |
| Self-Esteem | 49.84 (10.14) | 52.13 (8.24) | 51.22 (7.87) | 45.52 (12.45) | 45.95 (12.11) | **7.47 (< 0.001); 0.07**  **0.025 (NA > SUD)** | 0.07 |
| Self-Reliance | 47.72 (10.12) | 50.28 (8.62) | 49.17 (8.26) | 41.26 (13.18) | 41.38 (10.34) | **14.56 (< 0.001); 0.13**  **0.009 (NA > GD)**  **< 0.001 (NA > SUD)**  **0.037 (EG > GD)**  **0.002 (EG > SUD)** | 0.14 |
| Clinical Maladjustment | 49.50 (11.01) | 46.05 (9.06) | 47.04 (8.98) | 54.00 (11.01) | 58.11 (12.02) | **19.01 (< 0.001); 0.17**  **0.001 (NA < GD)**  **< 0.001 (NA < SUD)**  **0.017 (EG < GD)**  **< 0.001 (EG < SUD)** | 0.16 |
| School Maladjustment | 49.16 (10.44) | 45.58 (8.90) | 47.83 (9.61) | 53.78 (10.01) | 57.22 (10.81) | **18.97 (< 0.001); 0.17**  **< 0.001 (NA < GD)**  **< 0.001 (NA < SUD)**  **0.046 (EG < GD)**  **< 0.001 (EG < SUD)** | 0.15 |
| Personal Adjustment | 48.73 (10.78) | 51.65 (8.93) | 51.41 (7.99) | 40.41 (13.66) | 41.41 (12.32) | **19.20 (< 0.001); 0.17**  **0.001 (NA > GD)**  **< 0.001 (NA > SUD)**  **0.003 (EG > GD)**  **< 0.001 (EG > SUD)** | 0.19 |
| ESI | 50.77 (11.25) | 47.67 (8.83) | 48.65 (10.19) | 60.67 (15.56) | 56.35 (11.79) | **17.50 (< 0.001); 0.16**  **0.001 (NA < GD)**  **0.001 (NA < SUD)**  **0.005 (EG < GD)**  **0.013 (EG < SUD)** | 0.15 |
| Aggression | 44.36 (8.40) | 43.03 (7.74) | 44.97 (7.18) | 46.00 (9.06) | 47.93 (10.03) | **3.59 (0.014); 0.04**  **0.016 (NA < SUD)** | 0.04 |
| Hyperactivity | 45.24 (9.26) | 43.32 (8.31) | 45.14 (8.08) | 46.09 (10.81) | 46.69 (10.28) | 1.76 (0.156) | 0.02 |
| Conduct Problems | 46.77 (9.96) | 45.71 (7.97) | 42.44 (6.68) | 46.91 (12.66) | 54.17 (15.20) | **8.92 (< 0.001); 0.10**  **0.031 (NA < SUD)**  **0.002 (EG < SUD)** | 0.10 |
| Attention Problems | 47.72 (10.15) | 45.64 (9.82) | 47.53 (8.67) | 53.45 (12.01) | 51.03 (10.58) | **5.56 (0.001); 0.06**  **0.004 (NA < GD)**  **0.040 (NA < SUD)** | 0.06 |
| Atypicality | 48.09 (10.58) | 46.95 (10.02) | 46.11 (6.98) | 50.45 (10.46) | 51.72 (15.13) | 2.49 (0.61) | 0.03 |
| Depression | 45.71 (9.47) | 44.75 (8.49) | 42.47 (5.25) | 46.27 (8.58) | 48.97 (10.48) | **3.47 (0.017); 0.04**  **0.021 (EG < SUD)** | 0.04 |
| Anxiety | 47.87 (9.85) | 46.21 (8.91) | 46.94 (9.17) | 46.82 (8.54) | 52.34 (12.37) | **3.48 (0.017); 0.04** | 0.04 |
| Withdrawal | 50.37 (10.13) | 50.05 (9.27) | 51.17 (10.36) | 53.68 (11.96) | 51.21 (11.57) | 0.92 (0.433) | 0.01 |
| Somatisation | 47.91 (9.93) | 46.60 (8.31) | 47.14 (5.97) | 51.32 (13.13) | 53.14 (15.47) | **4.73 (0.003); 0.05** | 0.05 |
| Social Skills | 53.94 (9.62) | 54.70 (9.74) | 54.33 (8.83) | 52.36 (10.82) | 49.00 (11.58) | **2.84 (0.039); 0.03**  **0.026 (NA > SUD)** | 0.03 |
| Leadership | 52.72 (10.18) | 53.47 (9.77) | 52.81 (9.46) | 49.82 (7.71) | 51.38 (12.69) | 1.08 (0.358) | 0.01 |
| Externalising Problems | 45.74 (9.34) | 43.58 (8.13) | 45.19 (6.52) | 47.68 (11.23) | 51.00 (13.03) | **6.23 (< 0.001); 0.07**  **0.028 (NA < SUD)** | 0.07 |
| Internalising Problems | 46.57 (10.57) | 45.06 (8.21) | 44.56 (6.90) | 45.87 (14.84) | 52.28 (14.13) | **4.76 (0.003); 0.05**  **0.049 (EG < SUD)** | 0.06 |
| Adaptive Skills | 53.83 (10.08) | 54.66 (9.94) | 54.36 (9.68) | 51.18 (9.93) | 50.03 (12.62) | 2.17 (0.093) | 0.02 |
| BSI | 45.67 (10.41) | 43.60 (9.43) | 44.06 (7.52) | 47.82 (11.51) | 50.48 (13.18) | **4.66 (0.003); 0.05** | 0.05 |

*BSI, Behavioural Symptom Index; EG, Excessive Gaming; ES, Effect size; ESI, Emotional Symptom Index; GD, Gaming Disorder; M, average; n, sample; NA, no addiction; SD, standard deviation; SUD, Substance Use Disorder; Variables with significant ANOVA test results (p < 0.05) are shown in bold. Effect size = partial eta squared: 0.01 small, 0.06 medium, 0.14 large.*

**SUPPLEMENTARY TABLE 2 |** Unadjusted odds ratio of the multiple logistic regression (using no addiction as the reference category) to predict the dependent variables of Excessive Gaming and Gaming Disorder.

|  | EG | | GD | |
| --- | --- | --- | --- | --- |
| Independent Variables | OR  (95% CI) | *p*-value | OR  (95% CI) | *p*-value |
| Sex* | **17.368**  **(6.925–43.559)** | **< 0.001** | **4.321**  **(1.848–10.104)** | **0.001** |
| Adolescent Affection–Communication | 0.990  (0.966–1.015) | 0.414 | **0.957**  **(0.934–0.980)** | **< 0.001** |
| Mother’s Revoking Privileges | 1.744  (0.993–3.066) | 0.053 | **2.265**  **(1.185–4.329)** | **0.013** |
| Father’s Warmth | 0.997  (0.661–1.506) | 0.990 | **0.486**  **(0.274–0.861)** | **0.013** |
| Father’s Indifference | **1.810**  **(1.147–2.854)** | **0.011** | **2.597**  **(1.467–4.598)** | **0.001** |
| Father’s Reasoning | 1.276  (0.771–2.114) | 0.343 | **0.527**  **(0.286–0.973)** | **0.041** |
| Father’s Acceptance/Involvement | 0.776  (0.382–1.576) | 0.482 | **0.269**  **(0.115–0.628)** | **0.002** |
| Conscientiousness | 0.972  (0.939–1.007) | 0.112 | **0.901**  **(0.857–0.947)** | **< 0.001** |
| Openness | 0.990  (0.958–1.024) | 0.567 | **0.943**  **(0.904–0.984)** | **0.007** |
| Extraversion | 0.987  (0.956–1.020) | 0.447 | **0.942**  **(0.904–0.982)** | **0.005** |
| Agreeableness | 0.998  (0.964–1.034) | 0.921 | **0.897**  **(0.853–0.943)** | **< 0.001** |
| Neuroticism | 0.995  (0.966–1.025) | 0.747 | **1.054**  **(1.015–1.094)** | **0.006** |
| Negative Attitude Toward School | 1.012  (0.978–1.047) | 0.498 | **1.076**  **(1.036–1.117)** | **< 0.001** |
| Negative Attitude Toward Teachers | 1.029  (0.994–1.065) | 0.104 | **1.083**  **(1.041–1.127)** | **< 0.001** |
| Atypicality | 1.011  (0.971–1.053) | 0.597 | **1.054**  **(1.009–1.101)** | **0.018** |
| Locus of Control | 1.017  (0.981–1.054) | 0.363 | **1.090**  **(1.047–1.134)** | **< 0.001** |
| Social Stress | 0.999  (0.963–1.037) | 0.973 | **1.098**  **(1.059–1.139)** | **< 0.001** |
| Anxiety | 1.010  (0.979–1.043) | 0.530 | **1.057**  **(1.012–1.104)** | **0.013** |
| Depression | 1.008  (0.973–1.045) | 0.650 | **1.083**  **(1.049–1.119)** | **< 0.001** |
| Sense of Inadequacy | 1.015  (0.983–1.049) | 0.366 | **1.080**  **(1.041–1.119)** | **< 0.001** |
| Interpersonal Relationships | 0.992  (0.957–1.028) | 0.649 | **0.939**  **(0.910–0.969)** | **< 0.001** |
| Relationship with Parents | 1.009  (0.969–1.049) | 0.675 | **0.944**  **(0.915–0.974)** | **< 0.001** |
| Self-Esteem | 0.987  (0.951–1.025) | 0.503 | **0.938**  **(0.903–0.974)** | **0.001** |
| Self-Reliance | 0.986  (0.950–1.022) | 0.438 | **0.922**  **(0.887–0.958)** | **< 0.001** |
| Clinical Maladjustment | 1.012  (0.977–1.049) | 0.504 | **1.081**  **(1.038–1.126)** | **< 0.001** |
| School Maladjustment | 1.028  (0.992–1.065) | 0.133 | **1.091**  **(1.045–1.138)** | **< 0.001** |
| Personal Adjustment | 0.997  (0.959–1.036) | 0.865 | **0.920**  **(0.888–0.954)** | **< 0.001** |
| Emotional Symptom Index | 1.011  (0.977–1.047) | 0.516 | **1.092**  **(1.054–1.131)** | **< 0.001** |
| Conduct Problems | **0.945**  **(0.896–0.997)** | **0.037** | 1.016  (0.966–1.067) | 0.542 |
| Attention Problems | 1.020  (0.984–1.058) | 0.286 | **1.068**  **(1.026–1.112)** | **0.001** |

*CI, confidence interval; EG, Excessive Gaming; GD, Gaming Disorder; OR, odds ratio; Variables with significant ORs in the multiple logistic regression model (p < 0.05) are shown in bold; *Belonging to the male sex.*

**SUPPLEMENTARY TABLE 3 |** Odds Ratio adjusted by the sociodemographic variables in the multiple logistic regression model (using no addiction as the reference category) by applying a forward selection method (conditional) to predict EG and GD as dependent variables.

| Dependent  Variable | Independent variables | OR  (95% CI) | *p*-value |
| --- | --- | --- | --- |
| EG | **Sex*** | **17.368**  **(6.925–43.559)** | **< 0.001** |
| GD | **Sex*** | **4.321**  **(1.848–10.104)** | **0.001** |

*CI, confidence interval; EG, Excessive Gaming; GD, Gaming Disorder; OR, odds ratio. The variables in BOLD had a significant OR in the multiple logistic regression model (p < 0.05). *Belonging to the male sex category.*

**SUPPLEMENTARY TABLE 4 |** Odds Ratio adjusted by the TXP-A and TXP-C parenting questionnaire variables in the multiple logistic regression model (using no addiction as the reference category) by applying a forward selection method (conditional) to predict EG and GD as dependent variables.

| Dependent  Variable | Independent variables | OR  (95% CI) | *p*-value |
| --- | --- | --- | --- |
| EG | AAC | 0.990  (0.966–1.015) | 0.414 |
| GD | **AAC** | **0.957**  **(0.934–0.980)** | **< 0.001** |

*AAC, Adolescent Affection-Communication; CI, confidence interval; EG, Excessive Gaming; GD, Gaming Disorder; OR, odds ratio. The variables in BOLD had a significant OR in the multiple logistic regression model (p < 0.05).*

**SUPPLEMENTARY TABLE 5 |** Odds Ratio adjusted by the simple variables of the ESPA-29 questionnaires in the multiple logistic regression model (using no addiction as the reference category) by applying a forward selection method (conditional) to predict EG and GD as dependent variables.

| Dependent  Variable | Independent variables | OR  (95% CI) | *p*-value |
| --- | --- | --- | --- |
| EG | **Mother’s Revoking Privileges** | **2.174**  **(1.121–4.216)** | **0.022** |
|  | Father’s Warmth | 0.877  (0.51–1.496) | 0.631 |
| GD | **Mother’s Revoking Privileges** | **2.482**  **(1.017–6.058)** | **0.046** |
|  | **Father’s Warmth** | **0.339**  **(0.160–0.714)** | **0.004** |

*CI, confidence interval; EG, Excessive Gaming; GD, Gaming Disorder; OR, odds ratio. The variables in BOLD had a significant OR in the multiple logistic regression model (p < 0.05).*

**SUPPLEMENTARY TABLE 6 |** Odds Ratio adjusted by the global variables of the ESPA-29 questionnaires in the multiple logistic regression model (using no addiction as the reference category) by applying a forward selection method (conditional) to predict EG and GD as dependent variables.

| Dependent  Variable | Independent variables | OR  (95% CI) | *p*-value |
| --- | --- | --- | --- |
| EG | Father’s Acceptance/Involvement | 0.776  (0.382–1.576) | 0.482 |
| GD | **Father’s Acceptance/Involvement** | **0.269**  **(0.115–0.628)** | **0.002** |

*CI, confidence interval; EG, Excessive Gaming; GD, Gaming Disorder; OR, odds ratio. The variables in BOLD had a significant OR in the multiple logistic regression model (p < 0.05).*

**SUPPLEMENTARY TABLE 7 |** Odds Ratio adjusted by the BFQ-C questionnaire in the multiple logistic regression model (using no addiction as the reference category) by applying a forward selection method (conditional) to predict EG and GD as dependent variables.

| Dependent  Variable | Independent variables | OR  (95% CI) | *p*-value |
| --- | --- | --- | --- |
| EG | Conscientiousness | 0.957  (0.915–1.001) | 0.053 |
|  | Agreeableness | 1.026  (0.981–1.072) | 0.263 |
| GD | Conscientiousness | 0.943  (0.884–1.005) | 0.072 |
|  | **Agreeableness** | **0.932**  **(0.875–0.993)** | **0.030** |

*CI, confidence interval; EG, Excessive Gaming; GD, Gaming Disorder; OR, odds ratio. The variables in BOLD had a significant OR in the multiple logistic regression model (p < 0.05).*

**SUPPLEMENTARY TABLE 8 |** Odds Ratio adjusted by the simple variables of the BASC-S3 questionnaire in the multiple logistic regression model (using no addiction as the reference category) by applying a forward selection method (conditional) to predict EG and GD as dependent variables.

| Dependent  Variable | Independent variables | OR  (95% CI) | *p*-value |
| --- | --- | --- | --- |
| EG | Social stress | 1.000  (0.964–1.037) | 0.993 |
| GD | **Social stress** | **1.098**  **(1.059–1.139)** | **< 0.001** |

*CI, confidence interval; EG, Excessive Gaming; GD, Gaming Disorder; OR, odds ratio. The variables in BOLD had a significant OR in the multiple logistic regression model (p < 0.05).*

**SUPPLEMENTARY TABLE 9 |** Odds Ratio adjusted by the global variables (1) of the BASC-S3 questionnaire in the multiple logistic regression model (using no addiction as the reference category) by applying a forward selection method (conditional) to predict EG and GD as dependent variables.

| Dependent  Variable | Independent variables | OR  (95% CI) | *p*-value |
| --- | --- | --- | --- |
| EG | ESI | 1.011  (0.977–1.047) | 0.516 |
| GD | **ESI** | **1.092**  **(1.054–1.131)** | **< 0.001** |

*CI, confidence interval; EG, Excessive Gaming; ESI, Emotional Symptom Index; GD, Gaming Disorder; OR, odds ratio. The variables in BOLD had a significant OR in the multiple logistic regression model (p < 0.05).*

**SUPPLEMENTARY TABLE 10 |** Odds Ratio adjusted by the global variables (2) of the BASC-S3 questionnaire in the multiple logistic regression model (using no addiction as the reference category) by applying a forward selection method (conditional) to predict EG and GD as dependent variables.

| Dependent  Variable | Independent variables | OR  (95% CI) | *p*-value |
| --- | --- | --- | --- |
| EG | SMC | 1.031  (0.993–1.070) | 0.117 |
|  | PAC | 1.006  (0.966–1.049) | 0.764 |
| GD | **SMC** | **1.062**  **(1.012–1.115)** | **0.014** |
|  | **PAC** | **0.936**  **(0.901–0.973)** | **0.001** |

*CI, confidence interval; EG, Excessive Gaming; GD, Gaming Disorder; OR, odds ratio; PAC, Personal Adjustment; SMC, School Maladjustment. The variables in BOLD had a significant OR in the multiple logistic regression model (p < 0.05).*

**SUPPLEMENTARY TABLE 11 |** Odds Ratio adjusted by the BASC-P3 questionnaire in the multiple logistic regression model (using no addiction as the reference category) by applying a forward selection method (conditional) to predict EG and GD as dependent variables.

| Dependent  Variable | Independent variables | OR  (95% CI) | *p*-value |
| --- | --- | --- | --- |
| EG | **Conduct Problems** | **0.920**  **(0.867–0.976)** | **0.006** |
|  | **Attention Problems** | **1.050**  **(1.006–1.096)** | **0.025** |
| GD | Conduct Problems | 0.965  (0.909–1.025) | 0.246 |
|  | **Attention Problems** | **1.084**  **(1.033–1.137)** | **0.001** |

*CI, confidence interval; EG, Excessive Gaming; GD, Gaming Disorder; OR, odds ratio. The variables in BOLD had a significant OR in the multiple logistic regression model (p < 0.05).*
